# Supplementary material for: SE(3)-Equivariant Ternary Complex Prediction Towards Target Protein Degradation
Source: ArXiv. 2025 Feb 26:arXiv:2502.18875v1. Preprint. [Version 1] (PMC11888550)
Supplement: Supplement 1 [file NIHPP2502.18875v1-supplement-1.pdf]

Appendix A Supplementary Materials

A.1 Model selection on the validation set

For model selection during training, we evaluated model performance on a validation set comprising curated structures dissimilar to the training set but also not in the test set. After training, a single scalar Validation Performance Score (VPS) is calculated via a arithmetic mean of the DockQ scores for the top-ranked prediction based on PAE ($D_{\text{top-1}}$) and the best overall prediction (D_{best}):

$$\text{VPS} = (D_{\text{top-1}} + D_{\text{best}})/2 \tag{A1}$$

where D represents the DockQ score between the predicted and ground-truth crystal structures. Despite its simple definition, VPS assesses both structure and confidence accuracy.

The model's performance on the validation set is summarized in Supplementary Tab. A.1. Unlike the PROTAC, the performance on MG(D) plateaued with increasing seed numbers. To balance performance and computational cost, we used a single seed for MG(D) evaluation, resulting in VPS = $D_{\text{top-1}} = D_{\text{best}}$.

Supplementary Table A1 Results on the validation set for model selection. The highest VPS for each hyper-parameter is marked in bold. '=' denotes $D_{\text{best}} = D_{\text{top-1}}$ for MG(D)s, because only one seed is used for evaluation.

	PROTAC			MG(D)			Mean VPS
	$D_{\text{top-1}}$	D_{best}	VPS	$D_{\text{top-1}}$	D_{best}	VPS	
dim64	29.99	68.17	49.08	10.97	=	10.97	30.03
dim128	31.49	66.60	49.05	10.14	=	10.14	29.59
dim256	39.97	68.40	54.19	22.56	=	22.56	**38.37**
noise1	25.32	70.19	47.76	22.01	=	22.01	34.88
noise2	39.97	68.40	54.19	22.56	=	22.56	**38.37**
noise3	41.43	67.46	54.45	19.72	=	19.72	37.08
seed1	33.93	33.93	33.93	22.56	=	22.56	28.25
seed5	29.09	45.58	37.34	22.71	23.3	23.30	30.32
seed10	37.02	56.86	46.94	22.83	23.63	23.63	35.29
seed20	39.42	61.12	50.27	22.89	23.82	23.82	37.05
seed30	38.93	68.19	53.56	22.86	23.89	23.89	38.73
seed40	39.97	68.40	54.19	22.73	23.94	23.94	**39.06**

A.2 Reproducibility study

To assess the reproducibility of our method, we retrained the model five times using different random seeds (0, 1, 2, 3, and 4) for both model initialization and data sampling. Tab. A.3 summarizes the results, including mean, standard deviation, and range for each evaluated metric. Notably, the standard deviations are 3.50 for PROTAC top-1 DockQ, 1.36 for PROTAC best DockQ, and 1.47 for MG(D) top-1 DockQ. The largest variation was observed in the PROTAC top-1 DockQ metric, which ranged from 28.02 to 38.05. We also found that the RDKit version has a significant impact on performance; the results in Tab. A.3 were obtained using `rdkit==2023.9.3`.

A.3 Ligand pose accuracy

In the main manuscript, we reported the DockQ score for ternary complexes following established studies. Although the DockQ score only assesses protein-protein docking performance, it depends on accurate ligand positioning for the ternary complexes. For example, as shown in the manuscript Fig. 4f, complexes with the same protein pairs (e.g. PDB IDs 6W7O and 6W8I) can exhibit notable structural differences due to the variations in the PROTAC molecules. This observation is further supported by our analysis of BSAs in the BRD4-VHL and CRBN-BTK systems.

To directly evaluate ligand docking accuracy, we calculated the RMSD between the predicted ligand positions and their corresponding crystal structures. We report both the RMSD for the top-ranked prediction (based on predicted alignment error, PAE) and the best RMSD observed across all generated conformations. For PROTACs, the mean top-ranked RMSD is 3.43 Å, with 43% of predictions achieving an RMSD below 2 Å. By contrast, MG(D) predictions exhibit a significantly higher mean top-ranked RMSD of 13.14 Å, with only 1.9% of

Supplementary Table A2 Reproducibility study of model performance across five training runs with different random seeds. Reported are the mean, standard deviation, minimum, and maximum values for key performance metrics.

	PROTAC		MGD
	Top-1 DockQ	Best DockQ	Top-1 DockQ
Seed0	29.54	67.21	22.34
Seed1	28.02	65.73	18.89
Seed2	38.05	64.74	22.61
Seed3	31.22	66.23	21.85
Seed4	33.5	63.22	19.85
Mean	32.07	65.43	21.11
Std	3.50	1.36	1.47
Min	28.02	63.22	18.89
Max	38.05	67.21	22.61

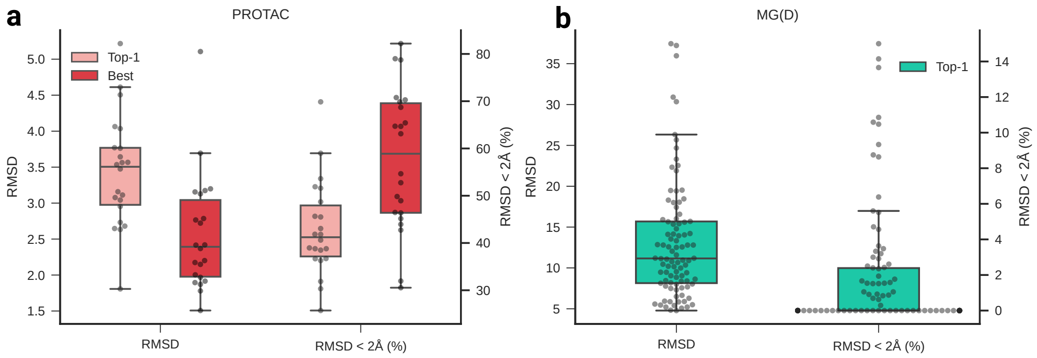

Supplementary Figure A1 Ligand pose accuracy on the test set of (**a**) PROTAC and (**b**) MG(D).

predictions falling below 2 Å. These results underscore the substantial challenge in accurately predicting MGD binding poses and highlight the need for further methodological development in this area.

A.4 Comparison with AlphaFold3 and Chai-1

During the submission period of this work, AlphaFold3 (AF3) [43] and Chai-1 [44] were proposed to tackle joint structure prediction, with both models capable of handling complexes involving proteins and small molecules. However, their training datasets differ from ours—AF3 was trained on PDB entries released before September 30, 2021, while Chai-1 used a cutoff of January 12, 2021—resulting in the inclusion of some of our test samples. To ensure a fair comparison, we filtered out PROTAC and MG(D) test clusters that were present in the training data of AF3 and Chai-1.

For PROTACs, three test complexes (7JTO_L_B_VKA, 7JTP_L_A_X6M, and 7Q2J_C_D_8KH) were not part of AF3's training set. We then performed MSA and template searches (to align with our unbound prediction setting as much as possible) and generated 40 predictions for each complex using different random seeds. The best DockQ scores for these three complexes were 0.12, 0.25, and 0.48 with AF3, compared to 0.56, 0.67, and 0.53 with our model (Tab. A3), indicating a clear decrease in performance of AF3. It is also worth noting that AF3 was trained on other 19 test complexes whereas our model never seen PROTAC-like structures during training. Furthermore, one complex (7KHH_C_D_WEP) was not included in Chai-1's training data; although Chai-1 achieved a high DockQ score of up to 0.81 on this complex, its performance on other PROTAC complexes was inferior.

For MG(D)s, all test set clusters were included in AF3's training data—either directly or through highly similar samples. As a result, AF3 achieved a high mean DockQ score of 0.52 on the MG(D) test set, based on one sampled random ligand conformation. There was only one MG(D) complex 7LRD_B_A_X5M that is not trained by Chai-1. For this complex, Chai-1 produced a DockQ score of just 0.009 compared to 0.014 from our method.

Supplementary Table A3 Comparison of DockQ with Chai-1 and AlphaFold3 (AF3) on the PROTAC test set. Our DeepTernary illustrates great generalization to unseen PROTAC and MG(D). – indicates that AF3 is trained on this complex cluster.

Type	Complex ID	Chai-1	AF3	DeepTernary
PROTAC	7JTP_L_A_X6M	0.40	0.25	**0.67**
	7JTO_L_B_VKA	0.04	0.12	**0.56**
	7Q2J_C_D_8KH	0.06	0.48	**0.53**
	7KHH_C_D_WEP	**0.81**	-	0.38
MG(D)	7LRD_B_A_X5	0.009	-	**0.014**

Supplementary Table A4 Comparison of SmRMSD on PROTACs. None indicates the item failed to generate an result.

PDB ID	Ignatov *et al.* [27]		Ours	
	SmRMSD	Best Rank	SmRMSD	Best Rank
5T35_D_A	2.01	1	0.61	3
5T35_H_E			0.70	1
6BN7_B_C	2.19	3	1.02	5
6BN8	2.21	1		
6BN9	1.35	1		
6BNB	1.63	5		
6BOY_B_C	5.24	3	2.94	4
6HAX_B_A	1.56	2	1.83	3
6HAX_F_E			1.68	3
6HAY_B_A	1.29	5	1.60	2
6HAY_F_E			1.35	5
6HR2_B_A	1.52	6	1.27	4
6HR2_F_E			1.68	3
6XHC	None	None		
6SIS_D_A			0.67	5
6SIS_H_E			0.84	2
6W7O_C_A			2.70	2
6W7O_D_B			1.12	2
6W8I_F_C			3.67	3
6W8I_D_A			1.34	2
6W8I_E_B			1.62	4
6ZHC_A_D			4.21	3
7JTO_L_B			2.62	5
7JTP_L_A	1.86	6	1.34	5
7KHH_C_D	2.41	4	4.63	3
7KHH_2				
7Q2J_C_D			2.59	1
7PI4	1.97	4.00		
Mean	2.12	3.36	1.91	3.18

These results indicate that despite the greater resources used to train Chai-1 and AF3, their poor performance on unseen PROTAC and MG(D) complexes highlights the superior generalization capability of our model.

A.5 PAE can be used as a confidence score for screening

Fig. A2 illustrates the correlation between DockQ scores and PAE values for both intra- and inter-complex predictions of PROTACs and MG(D)s. Overall, our results reveal a strong negative correlation—complexes with lower PAE scores tend to exhibit higher DockQ scores, reflecting more accurate predictions. Although several cases (e.g., 6BN7_B_C_RN3, 6BOY_B_C_RN6, 6HAX_B_A_FWZ, 6HAY_F_E_FX8, 6W7O_C_A_TL7, and 6ZHC_A_D_QL8) do not show a clear trend, the overall pattern supports the use of PAE scores as a reliable confidence metric and an effective filter for selecting high-quality predictions in drug discovery applications.

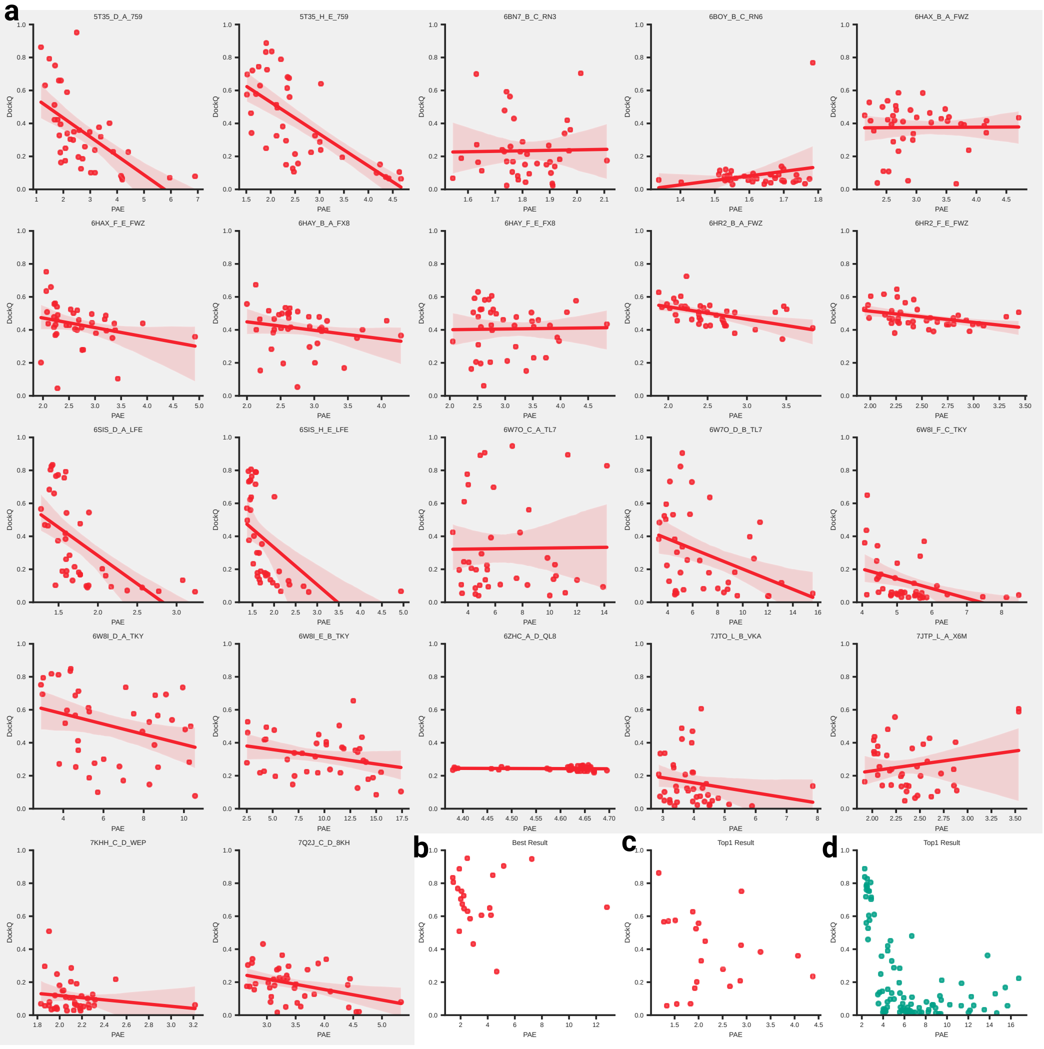

Supplementary Figure A2 Correlation between PAE and DockQ scores for predicted ternary complexes. a, Intra-complex correlation for PROTACs. Scatter plots illustrate the relationship between PAE and DockQ scores across 40 initial conformations for each PROTAC test complex. Each point represents a single conformation. Most complexes display a negative correlation, indicating that lower PAE values generally correspond to higher DockQ scores. This suggests that PAE can serve as a useful indicator of prediction accuracy within a given complex. **b,** Scatter plot for the best-predicted conformation (i.e., the one with the highest DockQ) for each PROTAC test complex. The plot demonstrates a clear trend: complexes with PAE scores below 4 tend to have higher DockQ scores (> 0.5), further supporting the use of PAE as a confidence metric. **c,** Across-Complex Correlation for top-1 PROTAC predictions (i.e., the predictions with the lowest PAE). Despite some false positives, the overall trend remains negatively correlated. **d,** Correlation for MG(D) predictions. Similar to PROTACs, a clear negative correlation is observed, with lower PAE values associated with higher DockQ scores, suggesting that PAE is also an effective confidence metric for MG(D) predictions.

Supplementary Table A5 Top-1 DockQ scores with different numbers of sampled random conformations on the PROTAC test set. The results are tested on the same checkpoint.

Sample Number	Top-1 DockQ
1	0.33
10	0.37
20	0.40
40	0.40

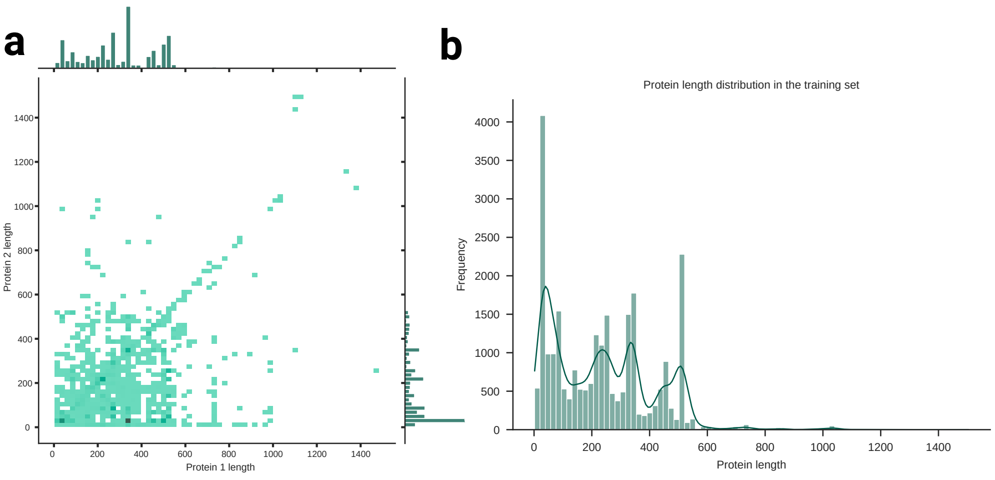

Supplementary Figure A3 Protein length distribution in the MG(D) training set. a, The joint distribution of sequence lengths for interacting chain pairs. **b,** The overall distribution of sequence lengths across all chains. The training set includes many proteins with sequence lengths between 200 to 600 residues, alongside a notable fraction of shorter proteins (20 - 40 residues). Additionally, the joint distribution reveals that the most common complex involves a longer protein interacting with a shorter protein–mirroring the characteristics of Group 2 complexes in the test set.

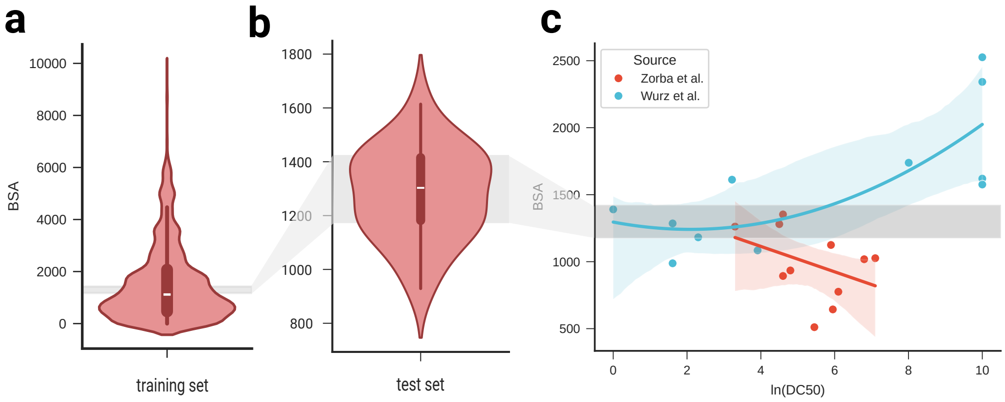

Supplementary Figure A4 Buried surface area (BSA) analysis of PROTAC-induced ternary complexes. a, BSA distribution in the training dataset, showing a peak around 1000 Å^2. **b,** BSA distribution for known PROTAC-induced complexes. **c,** Correlation between BSA and $ln(DC50)$ for BRD4-VHL and CRBN-BTK complexes, revealing an optimal BSA range for high degradation potential, rather than a linear relationship. The distinct BSA distribution in the training set compared to known complexes indicates the model learns generalizable principles beyond training set bias, suggesting its ability to predict degradation potential from predicted structures.

Supplementary Table A6 Hyperparameters of the model.

Hyperparameter	PROTAC	MG(D)
feature dim	256	256
encoder depth	8	8
decoder depth	1	4
number of pocket points	-	40
noise initial	2	2
batch size	64	64
optimizer	AdamW	AdamW
learning rate	1e-4	1e-4
weight decay	1e-4	1e-4
gradient clip	9	9
number of epochs	1000	1000

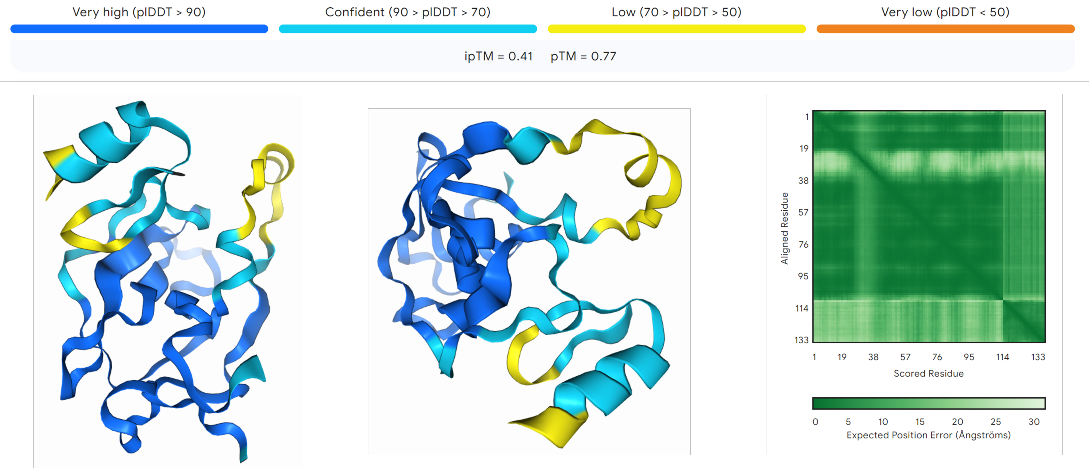

Supplementary Figure A5 Predicted structure of 7BQU_A_B from AlphaFold3, shown with pLDDT scores from two viewing angles. The Predicted Aligned Error (PAE) is displayed on the right.

Supplementary Table A7 DeepTernary Forward Pass Time. The execution times reported in the main manuscript include both the model's forward pass time and the time required for data preprocessing. This table specifically presents the forward pass time to allow for a more detailed analysis of model performance. *For PROTAC calculations, 40 conformations were used for each test sample.

Time (s)	w/o GPU	w/ GPU
PROTAC*	4.79	1.85
MG(D)	0.15	0.05
